# 4.D. Workshop: The interaction of environmental impact and chronic diseases: from theory to practice

**DOI:** 10.1093/eurpub/ckac129.218

**Published:** 2022-10-25

**Authors:** Organised by: EUPHA-CHR, EUPHA-ENV, Chair persons: Sarah Cuschieri (Malta), Marija Jevtic (EUPHA-ENV)

## Abstract

Both chronic diseases and environmental change are growing global public health challenges even amidst the ongoing Covid-19 pandemic. Every country is faced with these challenges, although small states and islands have an additional burden imposed on their sustainability, however this is rarely investigated. The intertwined relationship that exists between both epidemics is acknowledged, yet seldom explored or tackled together on a public health research level with bleak effect on policies outcome. People's lifestyles are a contributing factor to the progressive deterrent relationship between chronic diseases and environmental change. Indeed, many public health interventions promoting a healthier life will also produce the double benefit of tackling climate and the environment. For example, promoting more active transportation such as through the increase in bike lanes accessibility and incentives to promote active commuting will both reduce the dependency of carbon-based motor vehicle transportation, with a positive impact on the environment and climate, and on health. In fact, increase in physical activity is a well-known preventive and management action for most chronic diseases. Similarly, lower levels of pollution are also associated to decreases in many chronic diseases. Many other examples exist including the relationship between the food production, food security and carbon emissions. It is therefore imminent that urgent public health action is taken targeting the dominating urbanization and obesogenic environment that concomitantly have a role in the progressive development of chronic diseases and negative environmental impact. The aim of this workshop is to follow a multidisciplinary approach by bringing together different stakeholders to discuss the relationship between chronic diseases and the environment with a focus on small island states. While addressing the need to ensure that both public health and economic sustainability along with decelerating environmental change are on the imminent agenda. The workshop will be composed of four presenters discussing the various impacts on the environment from food production and security, sustainable dietetic models, the impact of city design on healthy living and finally the link between change in climate change and chronic diseases from a small state perspective. This will be followed by an engaging discussion between the presenters and the audience.

**Key messages:**

• An intertwined relationship exists between environmental change (ranging from food security to city planning) and chronic diseases.

• A One Health approach and public health interventions are required to halt the dual epidemic.

